# Mobile health and cardiac rehabilitation in older adults

**DOI:** 10.1002/clc.23306

**Published:** 2019-12-11

**Authors:** John Bostrom, Greg Sweeney, Jonathan Whiteson, John A. Dodson

**Affiliations:** ^1^ Department of Medicine New York University School of Medicine New York New York; ^2^ Rusk Department of Rehabilitation Medicine New York University Langone Health New York New York; ^3^ Leon H. Charney Division of Cardiology, Department of Medicine New York University School of Medicine New York New York; ^4^ Division of Healthcare Delivery Science, Department of Population Health New York University School of Medicine New York New York

**Keywords:** cardiac rehabilitation, home‐based cardiac rehabilitation, mobile health, older adults

## Abstract

With the ubiquity of mobile devices, the availability of mobile health (mHealth) applications for cardiovascular disease (CVD) has markedly increased in recent years. Older adults represent a population with a high CVD burden and therefore have the potential to benefit considerably from interventions that utilize mHealth. Traditional facility‐based cardiac rehabilitation represents one intervention that is currently underutilized for CVD patients and, because of the unique barriers that older adults face, represents an attractive target for mHealth interventions. Despite potential barriers to mHealth adoption in older populations, there is also evidence that older patients may be willing to adopt these technologies. In this review, we highlight the potential for mHealth uptake for older adults with CVD, with a particular focus on mHealth cardiac rehabilitation (mHealth‐CR) and evidence being generated in this field.

## 1 MOBILE HEALTH AND CHANGING DEMOGRAPHICS

By 2025, experts predict that 86% of the US population will be unique mobile phone service subscribers—with 91% of those owning smartphones—and there will be over 50 billion internet‐connected devices by 2020.^1^ It is currently estimated that 81% of all Americans own a smartphone.^2^ Mobile health (mHealth) is defined by the World Health Organization as the delivery of medical practice by mobile devices, including smartphones, tablets, or wearable monitoring devices^3^; this definition has more recently expanded to include mobile applications (“apps”), social media, and location tracking technology to obtain data relevant to surveillance, diagnosis, and management of chronic diseases.^4^

In the United States, cardiovascular disease (CVD) is the leading cause of death and accounts for the highest national direct health expenditure of any disease group.^5^ With demographic changes, the majority of patients suffering from CVD are older adults; for example, 60% of patients with chronic ischemic heart disease (IHD) are over the age of 65.^6^ By 2030, the number of adults older than 65 in the United States are expected to outnumber children for the first time in history, and accordingly, the prevalence of CVD is expected to rise.^7^ While younger people are widely reported to use mobile technology more frequently than older adults, the number of older adults using mobile technology is also increasing rapidly. Since 2011, the percentage of older adults who own a smartphone has almost quadrupled, and almost half of all Americans over the age of 65 now own a smartphone.^8^ Similarly, internet usage in older adults has increased considerably over the past 5 years, with almost two‐thirds of older adults reporting recent internet usage in 2016.^8^

With the ubiquity of mobile devices and internet connection, the scope and availability of mHealth have markedly increased in recent years. There are estimated to be more than 250 000 mHealth applications currently available to consumers, and many applications have been designed for surveillance and management of CVD. CVD, perhaps more than other disease domains, lends itself to synchronization with mHealth technologies, as many metrics relevant to disease management (heart rate, blood pressure, weight, rhythm analysis) are dynamic and quantifiable.^3^ To date, mHealth has been used to facilitate recovery after acute myocardial infarction (AMI),^9^ monitor arrhythmias,^10^ and to track ambulatory blood pressures.^11^ Applications have also been created to encourage medication adherence, facilitate social support,^3^ and augment the positive effects of cardiac rehabilitation (CR).

## 2 APPLICATIONS OF MHEALTH IN OLDER ADULTS: AN OVERVIEW

**Figure 1:**
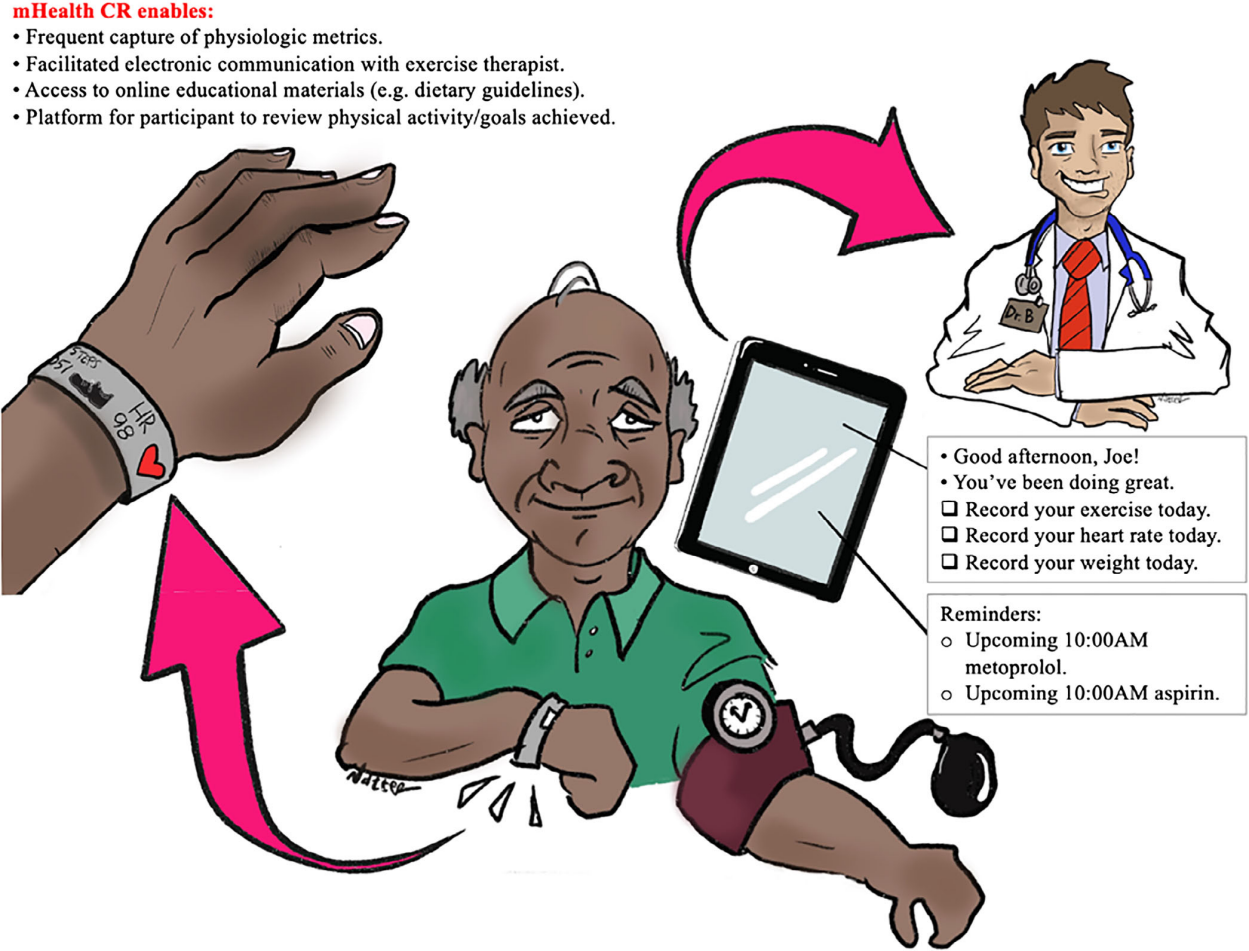
Role of mHealth‐CR in older adults

While a comprehensive review of the available modalities of mHealth is outside the scope of this review, there are several applications particularly relevant to older patients with CVD. Mobile applications, which are accessed via either by a smartphone or tablet, can allow for patients to actively input physiologic metrics, answer questions related to symptomatology, and access educational materials (Figure 1); numerous such applications have been developed for aid in the management of conditions including heart failure^12^, ^13^, ^14^, ^15^, ^16^ and hypertension.^11^, ^17^, ^18^ Smartphones themselves act as a passive sensor, as most are equipped with 9‐axis motion sensor and a 3‐axis accelerometer which can be used to track distance traveled, count steps, and even detect falls.^4^, ^19^ Such accelerometers have been used with some success in mobile applications that track patient activity in CR programs.^20^, ^21^

More recently, the increase in commercially available wearable devices (such as the Apple Watch [Apple, Cupertino, California]) has made arrhythmia detection possible by portable sensors. Wrist sensors, using photoplethysmographic monitoring (optically‐obtained measurements that detect changes in light related to blood flow in capillary beds, similar to those used for skin‐based pulse oximeters) can measure heart rate, heart rate variability, and use associated algorithms to detect arrhythmias including atrial fibrillation.^22^ Portable electrodes, such as the AliveCor Kardia Band (AliveCor, Mountain View, California), can also be connected to a wearable device and quickly generate a one‐lead electrocardiogram (ECG) to detect the presence of atrial fibrillation.^10^ Similar technology has not only been employed in the United States and Europe, but also lower‐resource settings such as rural India to detect atrial fibrillation.^23^

Improvement in medication adherence has been a target of mHealth development, and could be of particular use in older adults. Adherence can be assessed remotely via several mechanisms, including “E‐blisters,” or medication packets that send an electronic signal after being opened,^24^ or even pills can be embedded with a miniature sensor that emits a signal after entering the acidic environment of the stomach.^4^ With several mobile applications using mobile reminders for medication administration,^13^ there is opportunity for development of streamlined programs that potentially can decrease polypharmacy and confusion over home medication regimens while emphasizing medication adherence.

Patient education and empowerment with self‐monitoring is a potentially powerful tool offered by mHealth. mHealth interventions aimed at self‐monitoring of chronic conditions have shown improvement in reducing harmful behaviors such as smoking (albeit in younger populations).^18^ Two large ongoing studies will assess mHealth interventions aimed at improving self‐management in older populations. The SMArTVIEW trial (Self‐MAnagemenT‐VIsion for patient EmpoWerment), currently ongoing in the United Kingdom, will analyze whether Bluetooth enabled monitoring devices and mHealth‐delivered educational materials will optimize the ability of older adults following cardiac surgery to recognize when they require medical attention with hopes of improving postoperative outcomes.^25^ Also ongoing, the HATICE trial (Healthy Ageing Through Internet Counselling in the Elderly) will provide older adults at high risk for CVD with an interactive internet platform with educational materials and remote support by a coach with the goal of optimizing risk factors.^26^

As the population ages and CVD becomes more common, these types of mHealth applications could be of particular utility in older adults. A list of selected studies^11^, ^13^, ^14^, ^15^, ^16^, ^17^, ^18^, ^20^, ^21^, ^23^, ^24^, ^26^, ^27^, ^28^, ^29^, ^30^, ^31^, ^32^, ^33^, ^34^, ^35^, ^36^, ^37^, ^38^, ^39^ regarding mHealth in this domain that may be relevant to older adults is provided in Table 1. The remainder of this narrative review will focus on mHealth cardiac rehabilitation (mHealth‐CR) in older patients, which represents a particularly attractive target for intervention.

**Table 1:** Selected studies on mHealth in CVD

Author, name of study/technology (if applicable), year	Patient population, location	mHealth technology used	Key findings
Cardiac rehabilitation			
Worringham, CardioMobile, 2011^34^	6 patients, mean age 53, Australia	Smartphone application with single‐lead ECG and GPS‐tracking technology	This feasibility study offered an mHealth‐based CR program to a small group of patients who were unable to participate in traditional CR (following hospitalization for ACS or PCI). Participants showed improvement in 6‐minute‐walk test, reduced levels of depression, and improved QoL.
Antypas, 2014^39^	69 patients, mean age 59, Norway	Automated text messages to mobile phone, educational website	Intervention group received “tailored” mHealth approach involving automated text messages to mobile phone, questionnaires, and access to educational website that provided feedback based on patient responses. Compared to control group (traditional CR without text reminders), the mHealth group reported higher levels of physical activity 3 months after discharge from CR; there were no differences in self‐efficacy, social support, anxiety, or depression.
Forman, Heart Coach, 2014^21^	26 patients, mean age 59, USA	Mobile phone application	Mobile application prompted patients to complete a daily “task list” (including physical activity, medication reminders), provided educational material, tracked patient activity, and allowed CR providers to monitor patient progress. The app had favorable impact on adherence to CR, and older adults had no difficulty using the technology.
Varnfield, Care Assessment Platform, 2014^20^	120 patients, mean age 56, Australia	Mobile phone application with health diary, activity monitor, BP monitor, and scale	Patients randomized to smartphone‐based home CR program had significantly higher uptake, adherence, and completion than traditional CR. Both mHealth‐augmented and traditional groups showed similar improvements in 6‐minute walk assessment.
Maddison, HEART, 2015^38^	171 patients, mean age 60, New Zealand	Automated text messages to mobile phone, educational website	mHealth application used automated text messages multiple times per week to encourage home exercise and leisure‐time activity. Patients with IHD were randomized to mHealth‐augmented home CR vs usual community‐based CR; while there was no difference in peak oxygen uptake between groups, the mHealth group reported more leisure‐time physical activity and walking than the control group.
Prescott, EU‐CaRE, 2016^37^	1958 patients, age ≥65, Europe (Denmark, Netherlands, Italy, Germany)	Smartphone, HR monitor	Study ongoing; will enroll older patients who have declined traditional CR into an mHealth‐augmented home‐based CR and assess functional status and CR uptake at 12 months.
Widmer, 2017^40^	80 patients, mean age 63, USA	Online and smartphone‐based application	Patients randomized to mHealth‐augmented CR group (which provided educational materials and allowed for reporting of exercise and dietary habits) had significant improvements in weight loss and QoL; a nonsignificant reduction in CV‐related hospitalizations and ED visits (8.1% vs 26.6%, *P* = .054) was also seen.
Dodson, RESILIENT, 2019^36^	400 patients, age ≥70, USA	Tablet, eFitBit pedometer	Study ongoing; will randomize older patients to mHealth‐augmented CR and assess functional outcomes and health status at 3 months.
Heart failure			
Scherr, MOBITEL, 2009^16^	120 patients, mean age 66, Austria	Mobile phone application, internet program	Patients randomized to mHealth group had routine physiologic metrics (BP, weight) transferred to monitoring center for evaluation by physicians. In the per‐protocol analysis, patients in the mHealth group had fewer hospitalizations than usual care group (this difference was not seen in the intention‐to‐treat analysis).
Seto, 2012^27^	100 patients, mean age 54, Canada	Mobile phone application with Bluetooth connection to BP monitor, scale, ECG leads	Patients randomized to mHealth telemonitoring group (encouraged to record daily physiologic metrics through home vital sign measurements) reported improved QoL and self‐care scores; adherence was high in older patients. No difference between groups in mortality, ED visits, or hospitalization rates.
Layton, Wellframe, 2014^13^	16 patients, mean age 55, USA	Mobile phone application	Wellframe application provided medication reminders, educational materials (including information regarding smoking cessation), and tracked patient activity using phone pedometer; patients who were medically stable were more likely to use the application.
Vuorinen, Heart at Home, 2014^14^	94 patients with HFrEF, mean age 57, Finland	Mobile phone application	mHealth application allowed patients to self‐report physiologic metrics (weight, BP, HR) and answer questions regarding symptoms. There was no difference in number of HF‐related hospital days between usual care and mHealth group; mHealth group had more unplanned visits to nurses.
Comín‐Colet, iCor, 2015^15^	178 patients, mean age 77, Spain	Tablet with Bluetooth connection to BP monitor, scale; video conferencing	Patients randomized to mHealth group (with daily recording of symptoms, measurements of weight and BP, and scheduled videoconferencing with specialized HF program nurses) had reduced nonfatal HF events, HF readmissions, and CV readmissions. There was a 45% relative reduction in cost compared to usual care group.
Piette, CarePartner, 2015^28^	331 patients, mean age 68, USA	Interactive automated voice response calls, automated e‐mails	Patients were randomized to mHealth only vs mHealth with CarePartner groups. All patients received regular automated calls where they could self‐report HF symptoms, and reports would be sent to a clinician (all groups) as well as patient's self‐identified CarePartner (intervention group). Patients with a CarePartner reported increased medication adherence, and patients with baseline depressive symptoms were more likely to report positive assessments about their health.
Arrhythmia monitoring			
Skobel, HeartCycle, 2014^33^	50 patients, mean age 69, Germany	Shirt with ECG sensors	This validation study showed that a wearable ECG technology (shirt with ECG leads that measured HR, RR, and allowed for real‐time data reporting to physicians), had acceptable comparability for measurement of HR when compared to standard conventional cardiac exercise testing recordings.
Guo, mAFA, 2017^30^	209 patients, mean age 67, China	Mobile phone application	mHealth application provided clinical decision support tools and educational materials for patients. Patients who were randomized to mHealth intervention reported increased AF‐related knowledge, drug adherence, and anticoagulant satisfaction.
Mant, 2018^32^	120 000 patients age ≥65, England	Handheld single‐lead ECG device	This planned trial will randomize clinics across England to home AF screening vs no home screening, and follow outcomes (stroke, MI) over 5 years.
Steinhubl, mSTOPS, 2018^31^	2659 patients, mean age 72, USA	Portable ECG (iRhythm Zio) self‐applied patch	AF was diagnosed more frequently in the actively monitored group; anticoagulation was also prescribed more frequently in the actively monitored group. There was no significant difference in the number of AF‐related hospitalizations between groups.
Soni, SMART‐India, 2019^23^	2100 patients, mean age 61, India	Kardia AliveCor single‐lead ECG device	Population‐based AF screening study in rural India (Anand district, Gujarat) identified 1.6% of population with AF (three times higher than previously reported), with significantly higher rates of AF in older adults
Hypertension			
Kim, 2016^18^	95 patients, mean age 58, USA	Mobile phone, BP monitor	Patients randomized to mHealth group (wireless self‐monitoring of health behaviors, medication adherence, and BP monitoring) did not show a significant reduction in systolic BP.
Wijsman, iVitality, 2016^11^	151 patients, mean age 57, Netherlands	Mobile phone application, website, BP monitor	The iVitality application offered mobile reminders for patients to check and record their home BP; based on readings, patients were referred to an in‐person visit with their physician. Referred participants had a significant reduction in systolic blood pressure.
Morawski, MediSAFE‐BP, 2018^17^	412 patients, mean age 52, USA	Smartphone application	mHealth application included medication reminder alerts, adherence reports, and optional peer support. Patients randomized to mHealth group self‐reported higher medication adherence, though there was no difference in systolic BP compared to controls.
Medication adherence; self‐management			
Brath, mAMS, 2013^24^	53 patients, mean age 69, Vienna	Mobile phone application, e‐blisters	Through an mHealth application, patients could record when they were taking medications, and e‐blisters (packages that send an electronic signal once opened) recorded medication compliance. Patients randomized to mHealth group had increased adherence to oral diabetes medication (metformin); otherwise, no significant improvement in adherence was found.
Anglada‐Martínez, 2016^35^	48 patients, mean age 60, Spain	Mobile phone application, telemedicine	Older adults commonly refused an mHealth/telemedicine intervention (mobile application with educational materials aimed to improve adherence and patients' knowledge of their medication).
Richard, HATICE, 2016^26^	2600 patients, mean age ≥65, Netherlands, Finland, France	Interactive internet platform	Final results not yet reported. Intervention group provided with interactive internet platform providing educational materials and communication with a coach to facilitate self‐management of CV risk factors with follow‐up of 18 months. Primary outcome is composite of change in systolic BP, LDL, and BMI.

Abbreviations: ACS, acute coronary syndrome; ADL, activities of daily living; AF, atrial fibrillation; BMI, body mass index; BP, blood pressure; CR, cardiac rehabilitation; CV, cardiovascular disease; ECG, electrocardiogram; ED, emergency department; HF, heart failure; HFrEF, heart failure with reduced ejection fraction; HR, heart rate; IHD, ischemic heart disease; LDL, low density lipoprotein; mHealth, mobile health; MI, myocardial infarction; PCI, percutaneous coronary intervention; QoL, quality of life; RR, respiratory rate.

## 3 CR: BENEFITS AND CURRENT BARRIERS

CR, which is traditionally offered as a comprehensive center‐based program, has long been recognized as playing an important role in secondary prevention of events related to CVD, and is recommended by the American College of Cardiology (ACC) and American Heart Association (AHA) for use after acute myocardial infarction (AMI), percutaneous coronary intervention (PCI), and coronary artery bypass revascularization (CABG), as well as for chronic stable angina, and heart failure with reduced ejection fraction.^41^, ^42^ Accordingly, referrals to CR for all of the above diagnoses (as well as symptomatic peripheral arterial disease [PAD]) are reimbursed by the Center for Medicare and Medicaid Services.^43^, ^44^ CR reduces all‐cause mortality,^45^ cardiovascular mortality, and hospital readmissions, and improves health‐related quality of life and exercise capacity.^46^ Traditional CR is generally based at a single ambulatory center and involves a structured, supervised exercise program (usually 3 sessions per week for 36 total sessions) that are supervised by trained physicians, nurses, and exercise therapists.

Despite the known benefits of CR, referral and participation rates have remained stubbornly low. More than 80% of patients who are eligible for CR in the United States do not participate.^47^ Fewer than two‐thirds of patients who are eligible for CR are referred, and even when referred, only about one‐half will attend the first prescribed session.^48^ Certain populations, including older adults, are particularly under‐referred.^47^ Once enrolled, a substantial proportion of patients do not complete the prescribed 36 sessions; this is clinically relevant, as many studies suggest a dose‐dependent relationship between number of CR sessions attended and improved outcomes.^49^, ^50^ Older adults have several unique barriers to sustained participation in CR, including transportation issues (lack of a vehicle or vision/hearing impairment that precludes driving), cognitive impairment, and physical limitations (Table 2).^51^ These barriers and the aforementioned suboptimal referral and participation rates have been noted by the ACC/AHA, which have recommended that CR be “reengineered to include a wide array of service options that meet the needs of individual patients.”^47^

**Table 2:** Potential benefits and barriers to the adoption of mHealth‐CR

Potential benefits of mHealth‐CR	Potential barriers to use of mHealth‐CR
Improved accessibility to patients who are unable to attend traditional CR	Safety of mHealth‐based CR not yet well‐established
Ease of access to informational material regarding CR treatment plan	Physical limitations (eyesight, fine motor skills) may limit use in older adults
May improve engagement with CR treatment plan	Hesitance from older adults to adopt technology
Allows for more flexible CR schedule, patients can participate in sessions on their own	Less face‐to‐face interaction with CR clinical staff, and with other patients

## 4 NONTRADITIONAL CR

Home‐based CR has emerged as an alternative method of delivery to traditional center‐based CR programs. Home‐based CR involves prescribed exercise that can be carried out in a variety of settings and can be delivered “mostly or entirely outside of the traditional center‐based CR setting.”^52^ An option for decades in other countries (Australia, Canada), and even in some select health systems in the United States, home‐based programs have been evaluated by two recent Cochrane Reviews revealing no difference in outcomes (mortality, exercise capacity, cardiac events, or quality of life) when compared to traditional center‐based CR programs.^53^, ^54^ While some studies have shown that there is no difference in rates of adherence in home‐based vs traditional CR programs,^53^ others have suggested that patients may complete home‐based CR at higher rates.^55^ Patients, when given the choice between a home and center‐based CR program, often prefer a home‐based approach.^56^

There are several important differences between home‐based CR and traditional center‐based CR. Center‐based programs are located in medical facilities with groups of patients under direct in‐person supervision from physicians with access to emergency response capabilities. During their sessions, patients in traditional CR are typically monitored on telemetry. Home‐based CR programs, by definition, occur in settings other than healthcare facilities, and while no standardized home‐based CR program currently exists, patients typically are not directly supervised by medical staff, they are not monitored on telemetry, and they do not have the experience of exercising in groups. A recent ACC/AHA statement accordingly deemed home‐based CR a reasonable “alternative option to recommend for select clinically stable low‐to‐moderate risk patients who cannot attend traditional center‐based CR.”^52^ Data regarding the safety of home‐based CR have shown events are rare (one study estimates 1 event per 50 000 patient hours),^57^ but studies may have been underpowered to detect the risk of significant cardiovascular events in higher risk subgroups.^52^ HF‐ACTION, the largest trial to date involving hybrid home and center‐based CR, found no increased risk of cardiovascular events in the group prescribed at‐home exercise compared to traditional care.^42^ The recent HONOR trial, which evaluated an mHealth‐augmented home‐based exercise program for patients with PAD (in patients with a mean age of 70 years), also found no significant difference in significant adverse events between the intervention and control group.^58^ While further research is needed into the safety of home‐based CR programs, these results provide indirect support that they can be prescribed safely.

## 5 MHEALTH IN CR

Given that home‐based CR has been an option for years, the extension of mHealth to augment CR delivery represents a logical evolution in the broadening of its availability. Several studies, mostly outside of the United States, have used mHealth in creative ways to augment the CR experience. In an early study, Worringham and colleagues utilized a smartphone‐based program with a single‐lead ECG and GPS to employ a remote, walking‐based CR program for 134 monitored sessions in six patients in Australia, with data transmitted to an exercise therapist; patients using the program had similar improvements in 6‐minute walk test compared to those using traditional center‐based CR.^34^ A study of 62 male patients in Poland undergoing CR after acute MI also showed that a home‐based program, with remote ECG monitoring and a mobile device with preprogrammed exercise instructions, showed similar benefit in physical capacity compared to traditional CR.^59^ Several small studies have also supported the feasibility of various mHealth applications in the setting of CR, with success in monitoring medication adherence,^13^, ^21^ estimating levels of physical activity,^60^ and providing real‐time data to clinicians for feedback.^33^ Notably, older adults in these studies did not report difficulty using the mHealth technology.^21^, ^27^

Unfortunately, rigorous data regarding the effectiveness of mHealth in CR are still lacking. To our knowledge, one of the largest randomized‐controlled trial to date regarding mHealth in CR investigated 120 post‐AMI patients in Australia, randomizing patients to traditional CR vs a smartphone‐based home CR delivery model, with the smartphone application allowing for exercise monitoring, educational content delivery, and nutritional and psychological counseling via a web‐based portal. Results were promising; patients randomized to the intervention arm were more likely to participate in CR (80% vs 62%), had higher completion rates (94% vs 68%), and had similar positive exercise outcomes to the traditional CR group.^20^ A more recent trial in Minnesota comparing traditional CR and mHealth‐augmented CR also showed benefit; briefly, participants in the mHealth group received a smartphone or web‐based application that provided them with educational materials and allowed them to record their exercise and dietary habits (they were still encouraged to participate in the center‐based CR sessions). Participants in the mHealth group had significantly more weight loss, improved their diet, and reported higher quality‐of‐life scores than the control group at the completion of the CR program.^40^

The results of these trials, however, may not be generalizable. In the Australian study, participants were 87% male and had a mean age of 56 years^20^; in the US study, most were also male (82%) and relatively young (mean age 63).^40^ These trials therefore may have limited external validity when considering more typical “older adult” patients (commonly defined as age ≥75) who have both the highest CVD burden and the lowest familiarity with mHealth.

## 6 BARRIERS TO MHEALTH

Just as there are unique barriers for older patients to participate in traditional CR, there are potential barriers for participation with mHealth. While the use of mobile devices has become increasingly common, older adults have been slower to adopt newer technologies and devices than their younger counterparts.^61^ The Technology Acceptance Model, based on the Theory of Reasoned Action, states that the key factors for the adoption of a new technology are its perceived ease of use and its perceived usefulness.^62^ Perceived difficulty of use has in fact been cited as a reason for lack of use of mHealth applications by older patients,^3^ but other barriers exist: for example, age‐related sensory changes (fine motor skill deficiencies, vision loss) make certain devices more difficult to manipulate.^63^ The recent development of personal voice assistants (“Siri” by Apple; “Alexa” by Amazon), which interpret user voice commands, may be particularly useful in older populations, where vocalization is often maintained, and should be a focus in the development of mHealth applications going forward.^64^ While literature supports that older adults may accept new technologies, they often do so with less confidence than younger adults.^65^ In a qualitative study on the use of mHealth in older patients with heart failure, lack of knowledge, and even “fear” of misusing the technology, were the most commonly cited barriers to mHealth adoption.^66^

In addition to implementation barriers, there are other potential barriers to success: most notably, current mHealth‐CR platforms generally lack the socialization with other patients (peer support) inherent to facility‐based CR. This socialization, while difficult to quantify, may help to address the loneliness commonly experienced by older patients with CVD. “Virtual” peer‐to‐peer communication in mHealth‐CR platforms (eg, remote videoconferencing with others) may help to overcome this isolation, but to our knowledge this remains largely untested.

Another unknown factor is the comparative cost of mHealth vs facility‐based approaches to rehabilitation. Intuitively, mHealth approaches may be more cost effective as they enable delivery of care in the home environment, thus obviating the need for facility maintenance (and some studies have shown that mHealth interventions may be cost effective in the setting of HF and CR).^15^, ^38^ However, other costs related to mHealth (such as purchase of software and wearable devices) are highly variable.

## 7 FUTURE DIRECTIONS

For these reasons, it is imperative that further research into mHealth, and specifically mHealth‐CR, be performed in older adult populations. mHealth represents an opportunity to widen the scope of CR for those patients who would benefit the most from it. The RESILIENT trial (Rehabilitation Using Mobile Health for Older Adults with Ischemic Heart Disease in the Home Setting), now underway, is a prospective, randomized, NIH‐funded multicenter trial will employ an mHealth intervention to augment traditional CR in patients ≥70 years old. Participants in the intervention arm will be given mHealth‐CR software (through an electronic tablet device) that will allow for remote contact by an exercise therapist, and will be given activity trackers (eFitBit) to document their level of physical activity. Intervention (300 participants) and control groups (100 participants) will also be referred to traditional center‐based CR per ACC/AHA guidelines; the trial therefore represents a hybrid approach to CR. After 3 months, investigators will assess functional capacity (via 6‐minute walk test), goal attainment, health status, ability to perform instrumental ADLs, and hospital readmissions.^36^ The upcoming EU‐CaRE trial (European Study on Effectiveness and Sustainability of Current Cardiac Rehabilitation Programs in the Elderly) will evaluate 238 patients age ≥65 who have declined traditional CR and enroll them in an mHealth‐guided home‐based CR program with devices providing advice and coaching throughout the study period. Functional status (peak VO2), CR uptake, adherence, and participation will be recorded and compared to a traditional CR group (1720 patients).^37^ These trials, and others which are likely to take place, will be vital to the developing field of mobile CR, providing information regarding the safety and efficacy of mHealth‐augmented CR in older adults.

## 8 CONCLUSION

mHealth has enabled numerous avenues for remote management of CVD. Older adults, with the highest burden of disease, may stand to benefit the most. mHealth‐CR represents a particularly attractive area given traditional barriers to facility‐based CR. Small studies have demonstrated potential benefits to mHealth‐CR, but older adults have been under‐represented, and further research will help to elucidate engagement and outcomes among older adults who are prescribed this intervention.

## CONFLICT OF INTEREST

The authors declare no potential conflict of interest.
